# Elexacaftor/tezacaftor/ivacaftor influences body composition in adults with cystic fibrosis: a fully automated CT-based analysis

**DOI:** 10.1038/s41598-024-59622-2

**Published:** 2024-04-24

**Authors:** Dirk Westhölter, Johannes Haubold, Matthias Welsner, Luca Salhöfer, Johannes Wienker, Sivagurunathan Sutharsan, Svenja Straßburg, Christian Taube, Lale Umutlu, Benedikt M. Schaarschmidt, Sven Koitka, Sebastian Zensen, Michael Forsting, Felix Nensa, René Hosch, Marcel Opitz

**Affiliations:** 1 Department of Pulmonary Medicine, University Hospital Essen-Ruhrlandklinik, Essen, Germany; 2 Institute for Artificial Intelligence in Medicine, University Hospital Essen, Essen, Germany; 3 Institute of Diagnostic and Interventional Radiology and Neuroradiology, University Hospital Essen, Essen, Germany; 4 Adult Cystic Fibrosis Center, Department of Pulmonary Medicine, University Hospital Essen-Ruhrlandklinik, Essen, Germany

**Keywords:** Computed tomography, Nutrition

## Abstract

A poor nutritional status is associated with worse pulmonary function and survival in people with cystic fibrosis (pwCF). CF transmembrane conductance regulator modulators can improve pulmonary function and body weight, but more data is needed to evaluate its effects on body composition. In this retrospective study, a pre-trained deep-learning network was used to perform a fully automated body composition analysis on chest CTs from 66 adult pwCF before and after receiving elexacaftor/tezacaftor/ivacaftor (ETI) therapy. Muscle and adipose tissues were quantified and divided by bone volume to obtain body size-adjusted ratios. After receiving ETI therapy, marked increases were observed in all adipose tissue ratios among pwCF, including the total adipose tissue ratio (+ 46.21%, *p* < 0.001). In contrast, only small, but statistically significant increases of the muscle ratio were measured in the overall study population (+ 1.63%, p = 0.008). Study participants who were initially categorized as underweight experienced more pronounced effects on total adipose tissue ratio (p = 0.002), while gains in muscle ratio were equally distributed across BMI categories (p = 0.832). Our findings suggest that ETI therapy primarily affects adipose tissues, not muscle tissue, in adults with CF. These effects are primarily observed among pwCF who were initially underweight. Our findings may have implications for the future nutritional management of pwCF.

## Introduction

A poor nutritional status is linked to worse pulmonary function and increased mortality in people with cystic fibrosis (pwCF)^1,2^. Cystic fibrosis (CF)-associated poor nutritional status is a multifactorial syndrome caused by nutrient malabsorption, inadequate nutrient intake, decreased appetite and higher energy needs^3^. Therefore, clinical practice guidelines established therapeutic strategies for pwCF based on the individual nutritional status. For adults with CF, the recommended body mass index (BMI) is at or above 22–23 kg/m^21,3,4^. BMI is traditionally used to assess the nutritional status of pwCF since it is routinely calculated in the clinical practice. However, BMI is unable to distinguish between muscle, bone and fat mass and may miss pwCF with unfavorable body composition. A reduced fat-free mass index has been frequently found in pwCF with normal BMI^5^. In CF, higher fat-free mass has been significantly associated with improved pulmonary function and less exacerbations^6,7^. In recent years, CF transmembrane conductance regulator (CFTR) modulator treatment has significantly improved pulmonary function and quality of life in pwCF with a wide range of CFTR mutations^8–10^. Also an increase of BMI has been reported after treatment with the triple-combination CFTR modulator therapy elexacaftor/tezacaftor/ivacaftor (ETI), while there is limited evidence about the impact of ETI therapy on body composition in pwCF^5,11–14^.

So far, several techniques have been evaluated to assess the body composition in pwCF including dual-energy x-ray absorptiometry (DXA), bioelectrical impedance analysis (BIA), skinfold thickness and peripheral quantitative CT^5,15^. No accepted gold standard exists for monitoring the body composition in CF care as each method has its own advantages and disadvantages. Recent advances in deep-learning have led to the development of new methods for body composition analysis (BCA) that utilize a multi-resolution 3D U-Net for feature extraction^16–18^. These methods enable fully automated segmentation of volumetric body composition data on CT scans, replacing manual and semiautomatic segmentation methods. An in-house developed CT segmentation algorithm to calculate body composition features demonstrated strong correlations with BCA results obtained from DXA and BIA^16^. Furthermore, it exhibited performance at least comparable, if not superior, to an existing open-source segmentation algorithm^19,20^.

In this retrospective study, we used fully automated CT-based BCA to investigate the impact of CFTR modulator therapy on thoracic bone, muscle and adipose tissues in adult pwCF of our Cystic Fibrosis Center. Thereby, we hypothesized that adult pwCF receiving ETI therapy would experience specific effects on muscle and adipose tissues.

## Results

### Study population

The study population included 66 pwCF with a median age of 35 years and a range of 22–64 years at baseline (Table 1). Participating pwCF were either homozygous (38/66, 58%) or heterozygous (25/66, 38%) for the dF508 mutation. In addition, three pwCF with other CFTR mutations (3/66, 5%) received off-label treatment with ETI. Within the study cohort, 33/66 (50%) pwCF received prior mono- or dual-combination CFTR modulator therapy (ivacaftor, lumacaftor/ivacaftor or tezacaftor/ivacaftor). The median BMI was 19.5 kg/m^2^ (range 14.3–29.0 kg/m^2^), with 20/66 (30%) initially classified as underweight (BMI < 18.5 kg/m^2^) and 5/66 (8%) pwCF initially classified as overweight (BMI > 25 kg/m^2^). ETI therapy resulted in an increase of ppFEV1 (median + 10.5 points, + 28.38%, *p* < 0.001), an increase of BMI (median + 1.4 kg/m^2^, + 11.78%, *p* < 0.001) and a decline in sweat chloride levels (median − 54.5 mmol/L, − 54.90%, *p* < 0.001, Table 1). Patients with off-label ETI therapy showed comparable clinical responses (data not shown). After receiving ETI therapy, 5/66 (8%) pwCF were categorized as underweight and 9/66 (14%) as overweight (p = 0.003). Automated BCA was conducted in chest CT scans from all included study participants. CT scans were obtained 52 days (median) before starting on ETI. There were no significant changes in BMI between baseline CT scan and ETI initiation (p = 0.445). Most CT scans were performed as elective procedures for various reasons, such as monitoring of nontuberculous mycobacteria-/fungal-related lesions, investigating unexplained pulmonary function decline or screening for cancer before lung transplantation (53/66, 80%, Table 1). In 13/66 (20%) pwCF, chest CT scans were obtained due to acute clinical conditions, such as pulmonary exacerbation or hemoptysis. Follow-up chest CTs for BCA were performed 148–1147 days (median 529 days) after starting ETI therapy and 148–1191 days (median 675 days) after the initial CT scan. At follow-up, the reasons for CT scans did not differ from those at baseline, with the majority being elective procedures (Table 1).

**Table 1:** Characteristics of people with CF who received a CT-based body composition analysis at baseline and after initiation of ETI therapy.

n = 66	Baseline (T0)	ETI (T1)	*p* value
Age, years	35 (27.75; 42.5)		
Female sex, n (%)	31 (47)		
FEV_1_ (% predicted)	37 (28; 49.5)	47.5 (36.25; 63)	** < 0.001**
FEV_1_ [L]	1.35 (0.97; 1.9)	1.70 (1.22; 2.27)	** < 0.001**
BMI, kg/m^2^	19.5 (18; 21.95)	21.8 (20; 23.75)	** < 0.001**
Underweight, n (%)	20 (30)	5 (8)	**0.003**
Normal, n (%)	40 (61)	52 (79)	
Overweight, n (%)	5 (8)	9 (14)	
Body weight, kg	58 (52, 64)	62 (57, 72)	** < 0.001**
Body height, m	1.73 (1.65; 1.77)	1.72 (1.64, 1.77)	0.500
Body surface area, m^2^	1.68 (1.58; 1.79)	1.71 (1.61; 1.89)	** < 0.001**
Sweat chloride, mmol/L*	102 (92.5; 110)	46 (39; 63)	** < 0.001**
CFTR genotype, n (%)			
Homozygous dF508	38 (58)		
Heterozygous dF508	25 (38)		
Other**	3 (5)		
Prior CFTR modulator therapy, n (%)			
Mono-/dual-combination***	33 (50)		
None	33 (50)		
Chest CT scan			
Before/after ETI, days	52 (0;209.25)	529 (357.75; 648.25)	
Indication for CT scan, n (%)			0.999
Pulmonary exacerbation	10 (15)	9 (14)	
Hemoptysis	3 (5)	4 (6)	
Other (elective CT scan)****	53 (80)	53 (80)	

Values are median (first quartile, third quartile) or number of patients (%). P-value was determined using Wilcoxon signed rank test or Pearson Chi-squared test. * p < 0.05 ** p < 0.01 *** p < 0.001.CF, cystic fibrosis; FEV1, forced expiratory volume in 1 s; CFTR, cystic fibrosis transmembrane conductance regulator. *paired data available for 64 (pulmonary function, BMI, body weight) and 35 (sweat chloride) participants, respectively. **other: R553X/I336K, G542X/3849 + 10KbC- > T, R553X/3121-2A > G. ***mono-/dual-combination CFTR modulators: ivacaftor, lumacaftor/ivacaftor, tezacaftor/ivacaftor. ****reasons for elective CT scans: cancer staging, transplantation listing/cancer screening, monitoring of nontuberculous mycobacteria-/fungal-related lesions and allergic bronchopulmonary aspergillosis, fatigue of unknown reason, evaluation of pulmonary infiltrates, abnormal laboratory results, non-acute decline of pulmonary function or surveillance.

### Body composition analysis: baseline results

At baseline, adipose tissue ratios were strongly correlated with each other, with strongest correlations being between epicardial adipose tissue (EAT) and paracardial adipose tissue (PAT) ratios (r = 0.587, *p* < 0.001) and between subcutaneous adipose tissue (SAT) and intra- and intermuscular adipose tissue (IMAT) ratios (r = 0.878, *p* < 0.001, Figure S1). Muscle ratio and adipose tissue ratios showed no significant correlations (all p > 0.05). At baseline, total adipose tissue (TAT) ratio was higher among pwCF with prior CFTR modulator therapy, but these differences did not reach statistical significance (+ 37.73%, p = 0.054). IMAT and EAT ratios were found elevated among pwCF with prior baseline mono or dual-combination CFTR modulator therapy (+ 36.88%, p = 0.018; + 53.33%, p = 0.003; respectively). Muscle ratio was equally distributed between pwCF with and without CFTR modulator therapy at baseline.

### Body composition analysis: unadjusted longitudinal results

**Figure 1:**
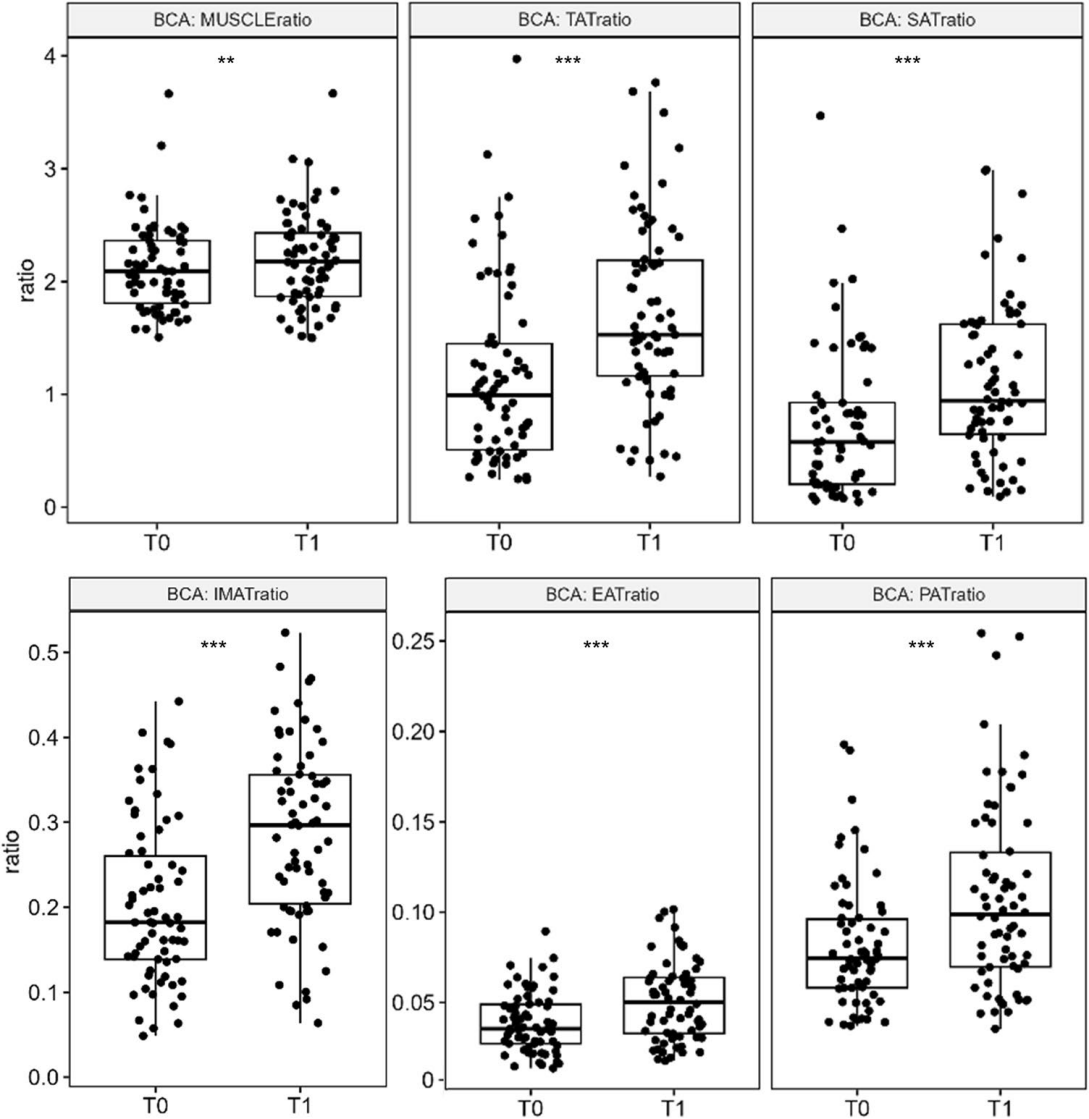
Unadjusted results from CT-based body composition analysis before (T0) and after (T1) elexacaftor/tezacaftor/ivacaftor therapy. Significant increases of muscle, IMAT, EAT, PAT, SAT and TAT ratios were observed between baseline (T0) and follow-up (T1) analysis of body composition. Bone volume was stable and utilized as the denominator for calculating body size-adjusted ratios. Statistics: Line at median. P-value was determined using Wilcoxon signed rank test. * p < 0.05; ** p < 0.01; *** p < 0.001. TAT, total adipose tissue; IMAT, intra- and intermuscular adipose tissue; EAT, epicardial adipose tissue; PAT, paracardial adipose tissue; SAT, subcutaneous adipose tissue.

Bone volume remained stable upon ETI therapy (Figure S2). BCA of follow-up CT scans demonstrated small, but significant increases of the muscle ratio in the overall study population (+ 1.63%, p = 0.008; Fig. 1). Changes in the muscle ratio and BMI showed significant correlations with improvements of ppFEV1 (r = 0.360, p = 0.004; r = 0.309, p = 0.015; respectively, Figure S3). One of the studied pwCF showed a significant decrease of muscle ratio (-26.41%, Figure S3B). This patient died as a result of acute-on-chronic liver failure and hemorrhagic shock three months after the follow-up CT scan. All adipose tissue ratios were significantly enhanced in response to ETI therapy, including increases of SAT (+ 50%, *p* < 0.001), IMAT (+ 34.62%, *p* < 0.001), EAT (+ 22.86%, *p* < 0.001), PAT (+ 21.62%, *p* < 0.001) and TAT ratios (+ 46.21%, *p* < 0.001, Fig. 1). Increases of TAT ratio and BMI showed strong correlation (r = 0.774, *p* < 0.001). A further analysis of the unadjusted data revealed more pronounced effects on adipose tissue ratios in pwCF who were initially classified as underweight (data not shown).

#### Body composition analysis: adjusted longitudinal results

**Figure 2:**
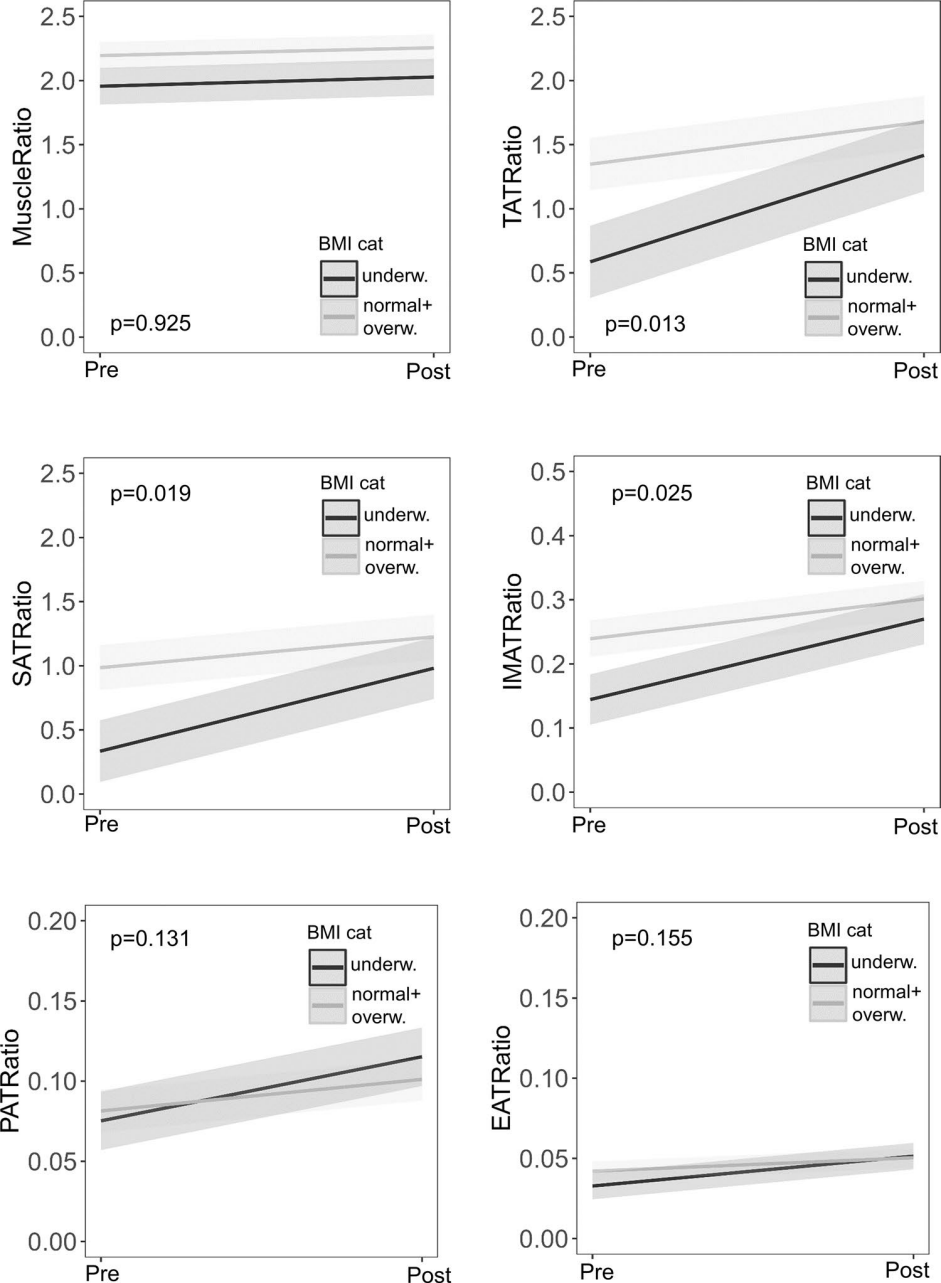
Adjusted results from CT-based body composition analysis (BCA) before (T0) and after (T1) elexacaftor/tezacaftor/ivacaftor (ETI) therapy. Estimated mean BCA outcome ratio (pre/post-ETI) by BMI category. Underweight pwCF (n = 20, BMI < 18.5 kg/m^2^) exhibited pronounced increase of TAT ratio (p = 0.013). Statistics: Generalized equation estimation models, estimated mean and standard deviation. P-value shown for interaction effect. Interaction effect time(pre/post)*BMI category adjusted for age at ETI start, biological sex and duration of ETI therapy. BMI, body mass index; TAT, total adipose tissue; IMAT, intra- and intermuscular adipose tissue; EAT, epicardial adipose tissue; PAT, paracardial adipose tissue; SAT, subcutaneous adipose tissue.

A multivariable regression analysis was performed to receive estimates for the mean change of BCA outcome parameters across BMI categories (underweight versus normal-/overweight at ETI start). Because of the medium-sized study population, outcomes were adjusted for a limited number of confounders, including biological sex, age at beginning of ETI therapy and duration of ETI therapy. The adjusted mean difference of TAT ratio was significantly higher among pwCF initially categorized as underweight (mean change of TAT ratio 0.831 versus 0.330, p = 0.013, Table 2, Fig. 2). In addition, SAT and IMAT ratios showed pronounced increases in pwCF who were initially underweight. In contrast, there was no impact of BMI category on change of muscle ratio (mean change of muscle ratio 0.072 versus 0.061, p = 0.925, Table 2, Fig. 2). Duration of ETI therapy was associated with higher muscle ratio (*p* < 0.001), but did not affect adipose tissue ratios. Male gender was associated with higher muscle ratios and lower TAT ratios in the overall study population (Table 2). However, ETI effects on muscle ratio and TAT ratio did not significantly differ between male and female pwCF (Figure S4).

**Table 2:** Adjusted multivariable analysis of TAT and muscle ratios. Adjusted mean difference (pre/post ETI therapy) shown for pwCF who were initially categorized as underweight (n = 20) versus normal- + overweight (n = 41 + 5 = 46).

Post versus Pre-ETI	Adjusted mean difference (95%CI)	p-value
Muscle ratio		
Interaction time (pre/post)*BMI category		**0.925**
**For BMI underweight**	**0.072 (**− **0.112, 0.255)**	**0.694**
**For BMI normal-/overw**	**0.061 (**− **0.151, 0.273)**	**0.847**
Age at ETI start, years	− 0.020 (− 0.024, − 0.015)	0.397
Biological sex, male	0.323 (0.211, 0.435)	< 0.001
ETI therapy, months	0.011 (0.005, 0.017)	< 0.001
TAT ratio		
Interaction time (pre/post)*BMI category		**0.013**
**For BMI underweight**	**0.831 (0.489, 1.173)**	**< 0.001**
**For BMI normal-/overw**	**0.330 (**− **0.097, 0.758)**	**0.174**
Age at ETI start, years	− 0.010 (− 0.020, 0.000)	0.053
Biological sex, male	− 0.740 (− 0.987, − 0.493)	< 0.001
ETI therapy, months	0.003 (− 0.011, 0.017)	0.659
SAT ratio		
Interaction time (pre/post)*BMI category		**0.019**
**For BMI underweight**	**0.647 (0.349, 0.944)**	**< 0.001**
**For BMI normal-/overw**	**0.240 (**− **0.135, 0.616)**	**0.315**
Age at ETI start, years	− 0.009 (− 0.017, − 0.001)	0.039
Biological sex, male	− 0.701 (− 0.917, − 0.485)	< 0.001
ETI therapy, months	0.002 (− 0.009, 0.013)	0.640
IMAT ratio		
Interaction time (pre/post)*BMI category		**0.025**
**For BMI underweight**	**0.125 (0.078, 0.173)**	**< 0.001**
**For BMI normal-/overw**	**0.062 (0.008, 0.116)**	**0.020**
Age at ETI start, years	− 0.002 (− 0.003, − 0.001)	0.024
Biological sex, male	− 0.061 (− 0.092, − 0.030)	< 0.001
ETI therapy, months	0.001 (− 0.001, 0.003)	0.227
EAT ratio		
Interaction time (pre/post)*BMI category		**0.131**
**For BMI underweight**	**0.019 (0.006, 0.032)**	**0.002**
**For BMI normal-/overw**	**0.008 (**− **0.001, 0,018)**	**0.111**
Age at ETI start, years	− 7.84e−5 (− 3.61e−4, 2.04e−4)	0.587
Biological sex, male	− 0.001 (− 0.008, 0.006)	0.833
ETI therapy, months	− 1.21e−4 (− 4,79e−4, 2.38e−4)	0.681
PAT ratio		
Interaction time (pre/post)*BMI category		**0.155**
**For BMI underweight**	**0.040 (0.014, 0.066)**	**0.001**
**For BMI normal-/overw**	**0.020 (**− **0.004, 0.043)**	**0.134**
Age at ETI start, years	− 0.001 (− 0.001, 0.000)	0.165
Biological sex, male	0.022 (0.008, 0.036)	0.002
ETI therapy, months	− 8.59e−4 (− 0.002, 9.75e−5)	0.078

Statistics: Values are estimates (95% confidence interval) calculated using generalized estimating equation models (GEE) for pre/post ETI outcome variables. CI, confidence interval; TAT, total adipose tissue; IMAT, intra- and intermuscular adipose tissue; EAT, epicardial adipose tissue; PAT, paracardial adipose tissue; SAT, subcutaneous adipose tissue. Bold text: Adjusted mean differences for pwCF with underweight versus normal/overweight BMI category.

## Discussion

Initiation of ETI therapy was associated with increased BMI in the phase 3 trial and real-world studies^8,11,12^. However, there is limited evidence on the impact of highly effective CFTR modulator therapy on changes in body composition^5^. In the present study, ETI therapy appeared to primarily affect adipose tissue ratios, particularly in pwCF with pre-treatment underweight.

At baseline, mono or dual-combination CFTR modulator therapy was associated with elevated IMAT and EAT ratios, but differences in the TAT ratio did not reach statistical significance. This study found no association between muscle ratio and mono or dual-combination CFTR modulator therapy at baseline. King et al. reported no significant differences in BMI, fat-free mass, or fat mass among pwCF with G551D mutation after 28 days of treatment with either ivacaftor or placebo. Significant increases in BMI and fat mass were observed at 5 months, with small gains in fat-free mass^21^. Likewise, Stallings et al. observed that ivacaftor therapy had a greater impact on fat mass than fat-free mass^22^. Another study analyzing 40 pwCF treated with lumacaftor/ivacaftor found significant increase in fat mass, but no significant increase in fat-free mass as measured by BIA^23^. Overall, these studies indicate that mono-/dual-combination CFTR modulators have more pronounced effects on adipose tissue/fat mass than muscle/fat-free (lean) mass.

In line with baseline data, our longitudinal analysis showed significantly increased adipose tissue ratios in pwCF receving triple-combination CFTR modulator therapy with ETI. All adipose tissues appear to be altered, with the most pronounced effects on the SAT ratio. However, we observed heterogeneous responses to ETI therapy. The highest gains in adipose tissue ratios were observed in individuals with underweight at ETI initiation, while pwCF with normal-/overweight experienced modest increases of adipose tissue ratios. Thus, baseline BMI category is the main determinant of the observed increases in the analyzed adipose tissues. Recent evidence showed that weight gain with ETI may not simply be attributed to increased energy intake^24^. Also, study participants did not routinely receive professional diet advice upon ETI initiation at our center. The mechanisms are likely multifactorial. Potential mechanisms of weight and adipose tissue gain are an improved intestinal fat absorption, reduced intestinal inflammation and decreased resting energy expenditure in pwCF receiving CFTR modulator therapy^22^. In addition, we observed small gains in the muscle ratio. Muscle gain correlated with improved pulmonary function. However, improved pulmonary function in response to ETI therapy was not limited to those with muscle gain, as those with stable or reduced muscle ratio also showed improvement. Hence, improved muscle ratio may be one, but not the only factor contributing to improved pulmonary function in pwCF treated with ETI. The marginal gains in muscle ratio were surprising. Improved pulmonary function and reduced airway obstruction might lead to less strain on thoracic muscles in pwCF. However, it is possible that the muscle ratio improved in other areas of the body not covered by chest CT scans. Moreover, there is no data on whether pwCF on ETI increase their physical activities or participate in training programs. We recently assessed health-related and skill-related components of physical fitness in a cohort of 21 pwCF after starting on ETI at our center and found no or marginal effects on physical fitness^25^. Current evidence on body composition in pwCF receiving triple-combination ETI is limited to a few studies. In a pilot study of 9 adolescents with CF receiving ETI therapy, Gur et al. recently reported both increased lean and fat mass using DXA^12^. Granados et al. reported increased weight and fat mass after ETI therapy in 8 pwCF, but no change of muscle mass as measured by DXA^13^. Another study using BIA found increases in fat and fat-free mass in 109 pwCF after starting ETI, with a higher percentage increase observed in fat mass^14^. Improvement of bone mineral density has been observed in a small cohort of pwCF receiving ETI^12^. Nevertheless, bone volume, as measured by automated BCA in this study, remained stable before and after ETI therapy and served as denominator to calculate body size-adjusted ratios. In the present study and in accordance with existing evidence, ETI therapy appears to result in imbalanced increases of muscle and adipose tissues. Individuals with underweight experience strong effects on adipose tissue ratios, while ETI therapy has a modest impact on muscle and adipose tissue composition in pwCF who are normal- and overweight. Individuals with normal or overweight BMI may benefit from close monitoring to prevent excess weight gain, especially in adipose tissue, upon receiving ETI therapy.

Despite being a frequent diagnostic tool in the care of pwCF, potentially valuable biometric information from CT scans often goes unused. Our work presents an approach to quantify all body tissues from routinely acquired CT scans, making BCA features useful for daily clinical practice. Automated CT-based BCA quantifies various tissue types and some might potentially serve as future biomarkers in CF care. We found strong correlations between IMAT and SAT ratios suggesting common tissue characteristics. In contrast, moderate correlation between SAT and EAT ratios may indicate differing physiology, as reviewed by Yim et al^26^. EAT has been thoroughly studied for its role in cardiovascular diseases and has distinct characteristics different from other adipose tissues. It directly contributes to the pathophysiology of coronary arteria disease through inflammatory processes and endothelial damage^27^. Thus, EAT could serve as a biomarker for cardiovascular risk in the ageing population of pwCF^28^. We observed a significant decline of muscle and TAT ratios in one patient, who later died due to hemorrhagic shock and acute-on-chronic liver failure. CT-based BCA might therefore have prognostic relevance and help to detect clinical decline at an early stage.

This exploratory study of body composition in pwCF has several limitations. First, CT-based BCA was performed in chest CT scans. Abdominal CT scans and/or full-body CT scans were not available and body composition might differ in other parts of the body. However, previous studies using manual and automated segmentation techniques of chest CT scans demonstrated the ability of chest CT scans to estimate the whole-body composition^29–31^. CT-based BCA was performed regardless of the indication for the CT scan. BCA parameters may or may not be influenced by short-term fluctuations in the patient´s clinical condition. Furthermore, our baseline cohort was heterogeneous regarding CFTR genotype and previous CFTR modulator therapy. In addition, the average ppFEV1 was lower than in the approval study for ETI. Therefore, our results might not be directly applicable to other cohorts. An untreated control cohort was not available as most pwCF were eligible for CFTR modulator therapy at our center. Hence, the natural course of body composition parameters during the study period remains unknown. As with other body composition methods, the minimal clinically important difference for body composition changes is not well established, particularly for rare diseases like cystic fibrosis. Further limitations are the monocentric and retrospective design of our study, which resulted in a variation of intervals between CT scans and ETI initiation. Future studies may benefit from a multicentric, prospective approach and a larger longitudinal cohort of pwCF to confirm our results and the overall applicability of automated BCA feature extraction in pwCF.

## Interpretation

Our results indicate that ETI therapy predominately affects adipose tissues and, to a lesser extent, muscle parameters in adult pwCF. These findings may have implications for the future nutritional management of pwCF. Fully automated CT-based BCA may be a valuable tool in the future, providing information on the individual nutritional status of pwCF each time a CT scan is performed.

## Methods

### Study population and ethics statement

**Figure 3:**
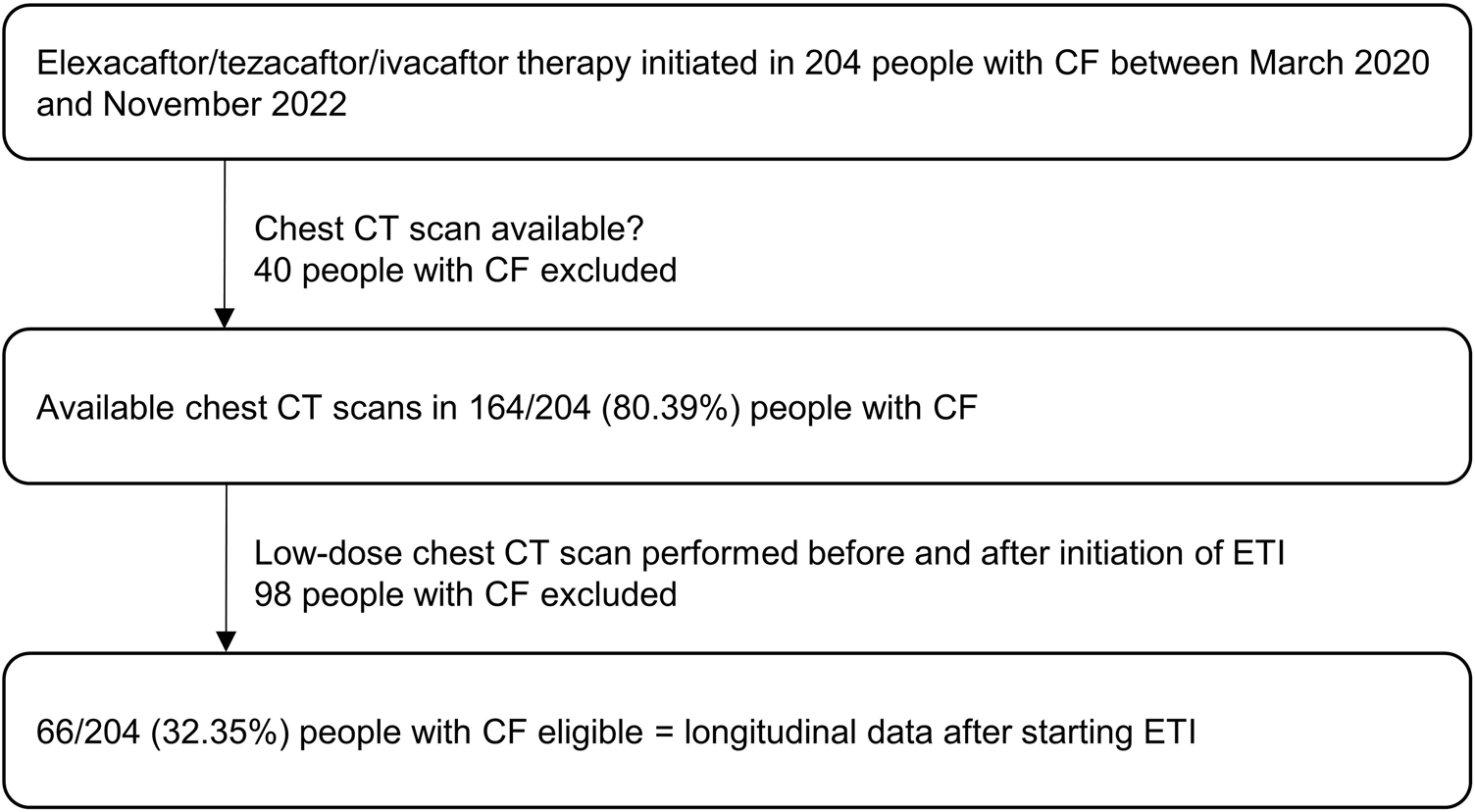
Study flow diagram showing inclusion criteria and analyzed cohort. Initially, 204 people with CF (pwCF) receiving elexacaftor/tezacaftor/ivacaftor (ETI) were screened. After filtering out 40 pwCF without available chest CT scans and 98 pwCF obtaining CT scans independent of ETI therapy initiation and/or with missing follow-up CT scan, 66 pwCF remained as the final cohort.

This retrospective, exploratory study analyzed the body composition of adults with CF beginning ETI therapy at our Cystic Fibrosis Center between March 2020 and June 2023. Patients were eligible for inclusion if they underwent a chest CT scan within 1 year before beginning ETI therapy (baseline data) and subsequently received a follow-up chest CT scan (longitudinal data), regardless of the indication for the CT scan (Fig. 3). Routine clinical data were extracted from medical records at baseline and during follow-up (BMI [kg/m^2^]; pulmonary function tests: percent predicted (pp) and absolute forced expiratory volume in 1 s (FEV1, [L]), sweat chloride levels [mmol/L]). Pulmonary function tests were performed after treatment of an impaired clinical condition at follow-up. The ethics committee of University of Duisburg-Essen approved this study (no. 22-11073-BO). The data was fully anonymized before being included in the study.

### CT-based body composition analysis

**Figure 4:**
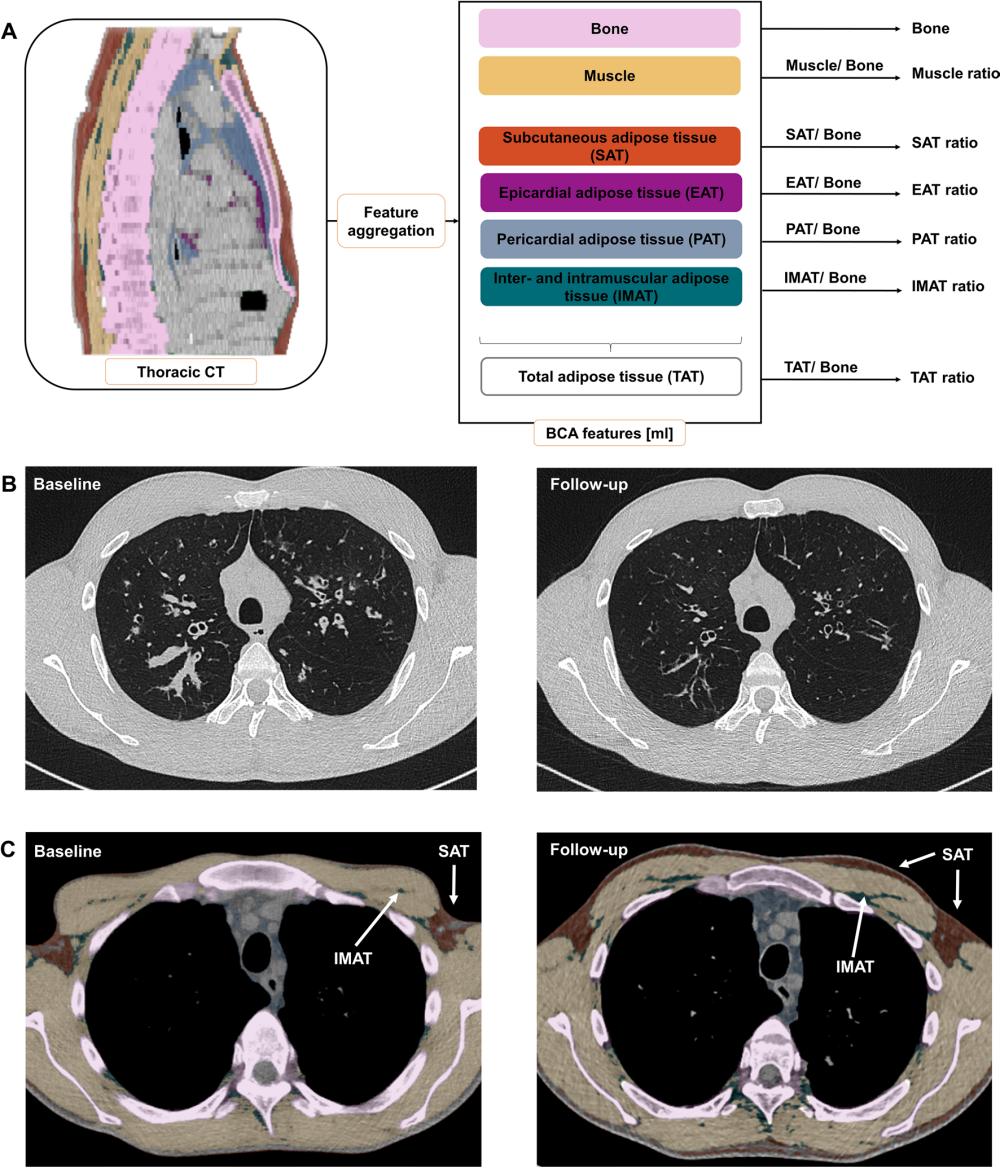
Exemplary in- and output of fully automated CT-based body composition analysis (BCA). (**A**) Visualization of feature extraction for BCA and marker aggregation. BCA network detects the different BCA features within the chest CT scan. Those raw features are combined with bone to calculate body size-adjusted biomarkers. The tissues are encoded in colors as follows: pink: bone, yellow: muscle, orange-brown: subcutaneous adipose tissue (SAT), purple: epicardial adipose tissue (EAT), light blue: paracardial adipose tissue (PAT) and turquoise: inter- and intramuscular adipose tissue (IMAT). (**B**) Exemplary chest CT in axial view before (left) and after (right) elexacaftor/tezacaftor/ivacaftor therapy showing decreasing bronchiectasis wall thickening and regredient mucus impaction. (**C**) Exemplary chest CT in axial view showing increased proportion of subcutaneous adipose tissue (SAT, orange-brown) and increased inter- and intramuscular adipose tissue (IMAT, turquoise) after elexacaftor/tezacaftor/ivacaftor therapy.

Low-dose non-contrast-enhanced chest CT scans were acquired using a 64 detector row single-source CT scanner (SOMATOM Definition AS, Siemens Healthcare GmbH, Forchheim, Germany) with a gantry rotation time of 300 ms (collimation: 64 × 0.6 mm, slice thickness: 5.0 mm, tube current time product: 25 mAs, tube voltage: 120 kV). Convolution kernels B31F and B70F were used in image reconstruction. To optimize radiation protection, automatic tube current modulation (CARE Dose4D, Siemens Healthcare GmbH, Forchheim, Germany) and automatic tube voltage selection (CARE kV algorithm, Siemens Healthcare GmbH, Forchheim, Germany) were applied. Patients were scanned in head-first-supine position with elevated arms and in inspiratory breath-hold. All scans were resampled to a slice thickness of 5.0 mm for use in the body composition network (Fig. 4B, C). The chest CT-based body composition features were extracted by a pre-trained deep-learning network, which is an evolution from the system described by Koitka et al.^18^. It employs a multi-resolution 3D U-Net architecture variant and enables fully automated segmentation of tissues within detected body regions in CT scans. The BCA was initially trained with the 3D U-Net network using a dataset comprising 300 CT scans, including both non-contrast and contrast-enhanced CT scans. Thereupon, the segmentation efficiency was evaluated on separate test-datasets which were annotated manually by experienced examiners^16,18^. Results from automated BCA, based on routine whole body CT scan in patients with neuroendocrine tumors, revealed strong correlations between body fat ratio measured with DXA and BIA, as well as between muscle ratio measured with BIA^16^. In comparison with the open-source automated segmentation tool TotalSegmentator, automated BCA achieved a comparable average Sørensen-Dice score (0.935), representing the accuracy of the results, and a higher mean voxel body coverage^16,19^. So far, automated BCA has been evaluated for various clinical applications at our center^16,32–34^. It has been made available to clinicians via an integration as a DICOM node at our center. Thus, imaging data remained within the organization. In this study, BCA quantified the volume [mL] of four different adipose tissue biomarkers: subcutaneous adipose tissue (SAT), intra- and intermuscular adipose tissue (IMAT), epicardial adipose tissue (EAT), and paracardial adipose tissue (PAT). The sum of the four adipose tissue markers was displayed as total adipose tissue (TAT). Raw BCA features were divided by bone volume to receive body size-adjusted ratios of muscle and adipose tissue measurements (Fig. 4A).

### Statistical analyses

GraphPadPrism 9 (GraphPad Software LLC, Boston, MA, USA) and R (R studio version 2023.09.1) were used for statistical operations. The Shapiro–Wilk test was used to test the normality of data. Continuous variables are shown as mean and standard deviation or median with first and third quartile, as indicated. Binary and categorical variables are presented as counts and percentages. Non-normal data were analysed using the Mann–Whitney U-test for unpaired data or the Wilcoxon signed-rank test for paired data. Pearson Chi-squared test or Fisher-exact test were used to assess frequency distributions of categorical data. Correlations were analyzed using the pairwise Spearman correlation test and visualized as correlation matrix. The generalized estimating equation (GEE) approach, applicable to non-normal and longitudinal data, was used to conduct a multivariable regression analysis. The mean differences between BCA outcome parameters before and after ETI therapy were estimated and stratified by BMI category (underweight versus normal-/overweight) using an interaction term between BMI category and time (pre/post ETI therapy). Thereby, potential differences in outcome parameters between BMI categories were tested. The model was adjusted for biological sex, age and duration of ETI therapy which differed by study participant. Statistical significance for all analyses was defined as *p* < 0.05.

### Supplementary Information

Supplementary Information.

## Data Availability

The data analyzed in this study is not publicly available. Data can be made available upon reasonable request and review by the local institutional review board. Requests may be sent to the corresponding author.

## Abbreviations

BIA: Bioelectrical impedance analysis
BCA: Body composition analysis
BMI: Body mass index
CF: Cystic fibrosis
CFTR: Cystic fibrosis transmembrane conductance regulator
DXA: Dual-energy x-ray absorptiometry
ETI: Elexacaftor/tezacaftor/ivacaftor
EAT: Epicardial adipose tissue
FEV1: Forced expiratory volume in 1 s
IMAT: Intra- and intermuscular adipose tissue
pwCF: People with cystic fibrosis
PAT: Paracardial adipose tissue
SAT: Subcutaneous adipose tissue
TAT: Total adipose tissue
