# Supplementary material for: Elexacaftor/tezacaftor/ivacaftor influences body composition in adults with cystic fibrosis: a fully automated CT-based analysis
Source: Sci Rep. 2024 Apr 24;14:9465. doi: 10.1038/s41598-024-59622-2 (PMC11043331; doi:10.1038/s41598-024-59622-2)
Supplement: Supplementary file 1 — Supplementary Information. [file 41598_2024_59622_MOESM1_ESM.docx]

**Online data supplement**

**Figures**


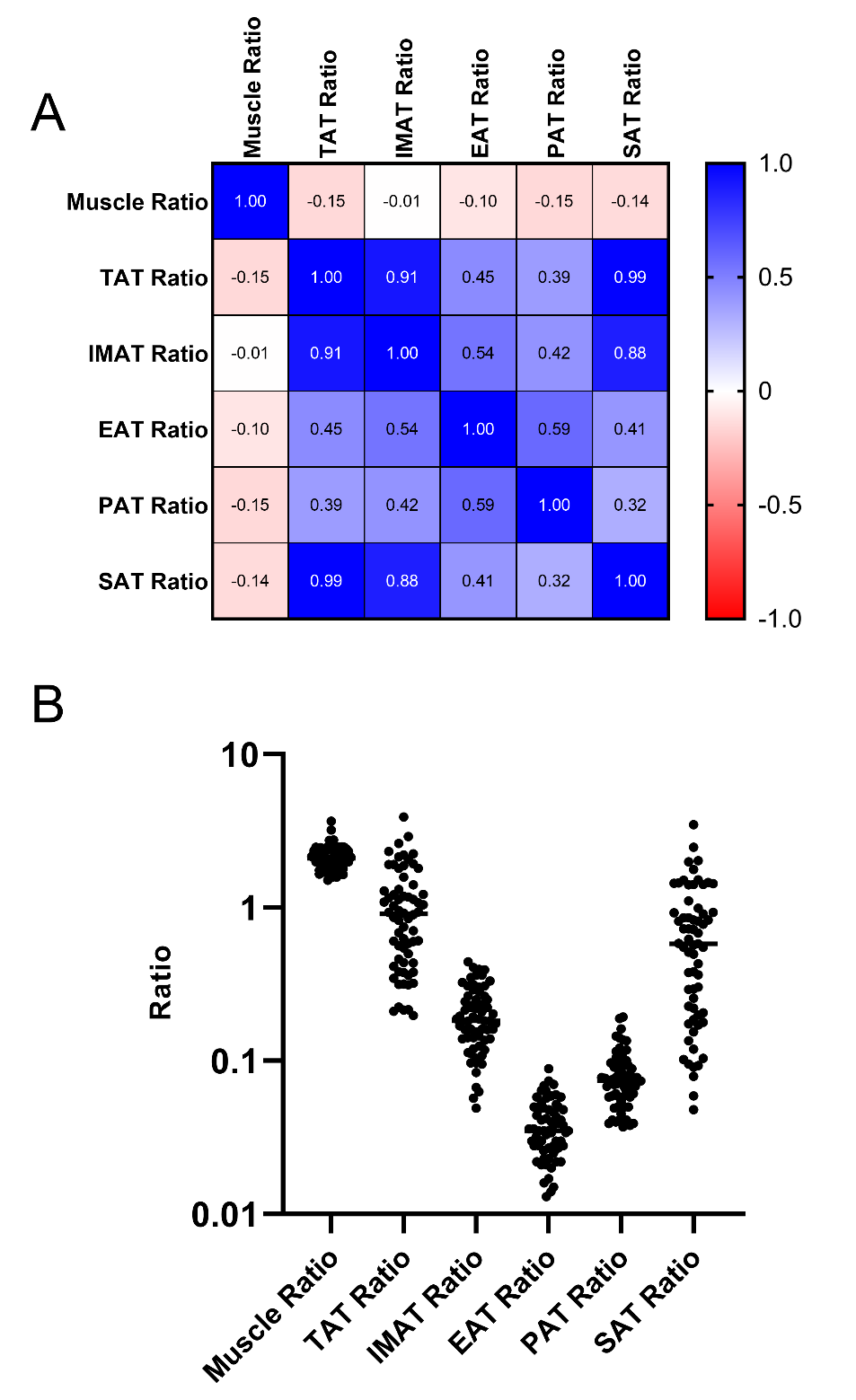


Figure S1: Baseline (T0, n=66) results from body composition analysis (BCA). A: Correlation matrix of BCA results. Muscle ratio did not significantly correlate with adipose tissue results. Adipose tissue ratios were strongly correlated with each other, with strongest correlations being between EAT and PAT ratio and between SAT and IMAT ratio. B: Distribution of BCA ratios at baseline. Statistics: Spearman correlation. IMAT, intra- and intermuscular adipose tissue; EAT, epicardial adipose tissue; PAT, paracardial adipose tissue; SAT, subcutaneous adipose tissue; TAT, total adipose tissue.


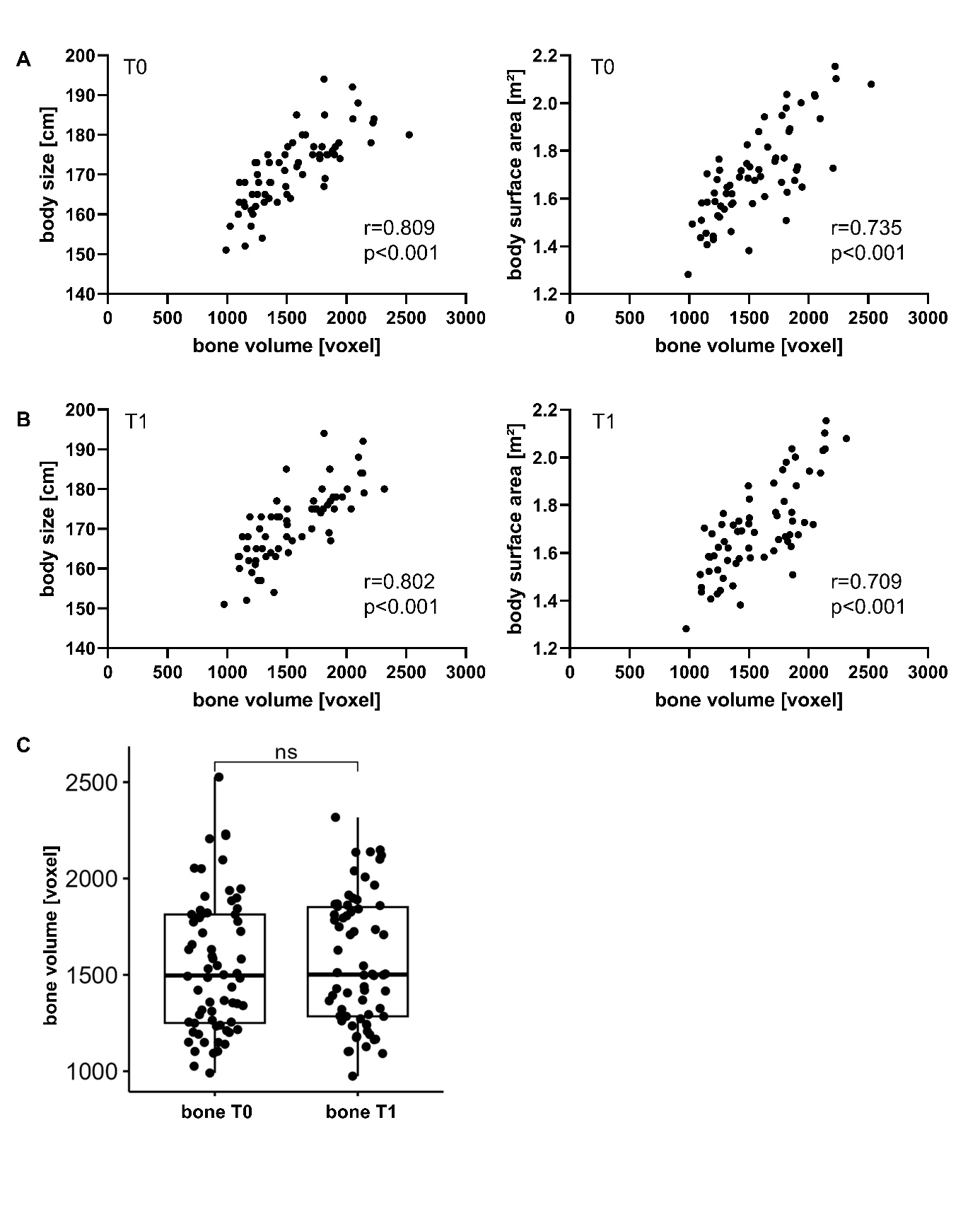


Figure S2: Results from CT-based body composition analysis (BCA) on bone volume. A: At baseline (T0), bone volume showed robust correlations with body size and body surface area (Du Bois method). B: At follow-up (T1), robust correlations between bone volume and body size/ body surface area (Du Bois method) remained. C: Bone volume, as measured by BCA, was stable before and after ETI therapy. Statistics: Line at median. Spearman correlation (A, B) and Wilcoxon signed rank test (C).

**
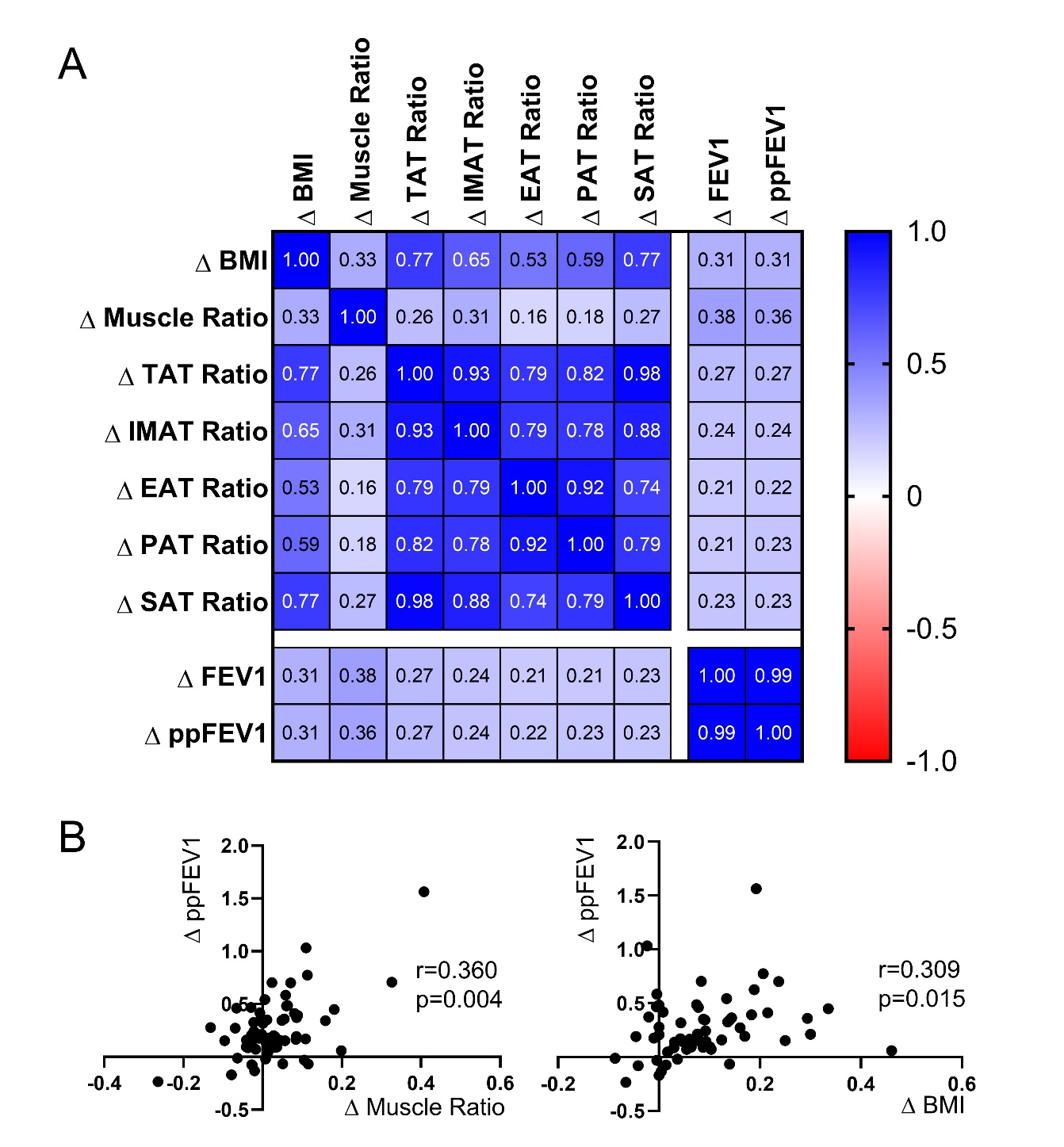
**

Figure S3: Changes of body composition parameters in response to elexacaftor/ tezacaftor/ ivacaftor therapy. A: Correlation matrix of delta change in pulmonary function (forced expiratory volume in 1 second [L, FEV1] and percent predicted [pp] FEV1) and delta change of body composition ratios. B: Correlation plots of delta change in ppFEV1 and delta change of muscle ratio/ delta change of BMI. Statistics: Spearman correlation. IMAT, intra- and intermuscular adipose tissue; EAT, epicardial adipose tissue; PAT, paracardial adipose tissue; SAT, subcutaneous adipose tissue; TAT, total adipose tissue; FEV1, forced expiratory volume.


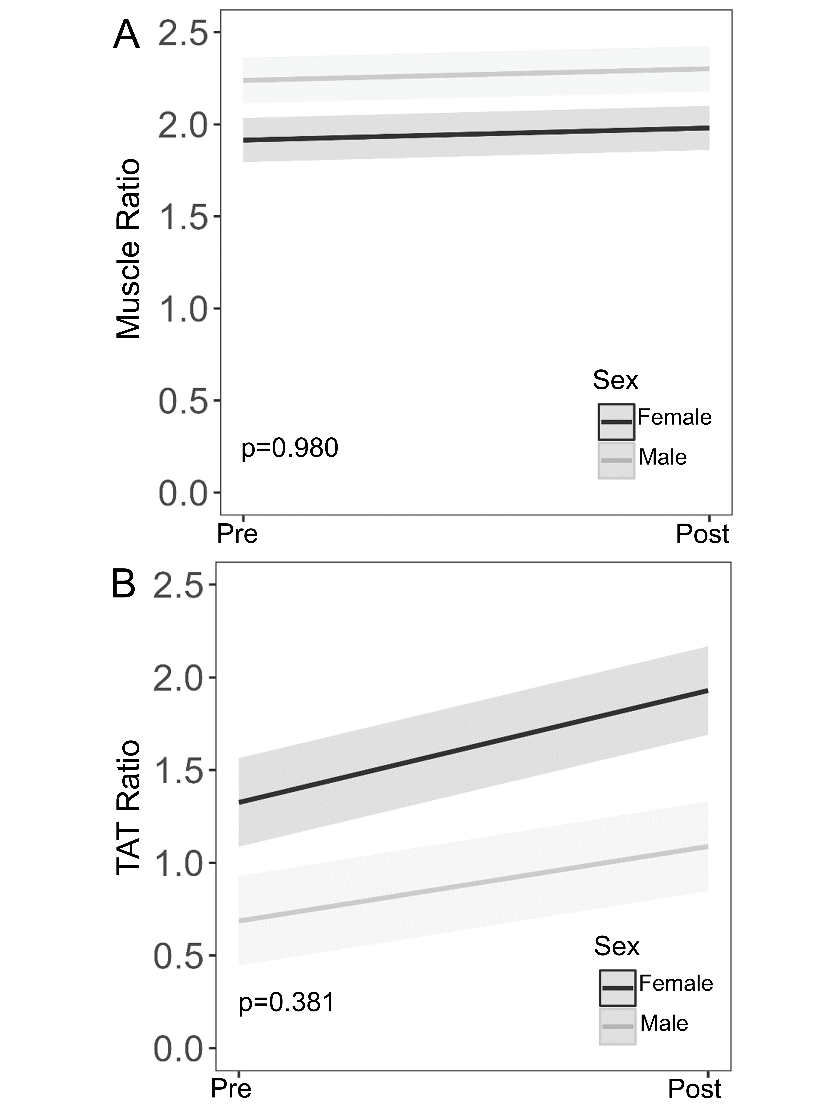


Figure S4: Adjusted results from CT-based body composition analysis before (T0) and after (T1) elexacaftor/ tezacaftor/ ivacaftor (ETI) therapy. A: Estimated mean muscle ratio (pre/post-ETI) by biological sex. B: Estimated mean TAT ratio (pre/post-ETI) by biological sex. Statistics: Generalized equation estimation model, estimated mean and standard deviation. P-value shown for interaction effect. Interaction effect time (pre/post)*biological sex adjusted for age at ETI start, BMI at start and duration of ETI therapy. TAT, total adipose tissue.
